# Role of BDNF Signaling in Memory Enhancement Induced by Transcranial Direct Current Stimulation

**DOI:** 10.3389/fnins.2018.00427

**Published:** 2018-06-26

**Authors:** Sara Cocco, Maria V. Podda, Claudio Grassi

**Affiliations:** ^1^Institute of Human Physiology, Università Cattolica del Sacro Cuore, Rome, Italy; ^2^Fondazione Policlinico Universitario A. Gemelli IRCCS, Rome, Italy

**Keywords:** tDCS, memory, synaptic plasticity, BDNF, epigenetics, personalized medicine

## Abstract

In the recent years numerous studies have provided encouraging results supporting the use of transcranial direct current stimulation (tDCS) as non-invasive brain stimulation technique to improve motor and cognitive functions in patients suffering from neurological and neuropsychiatric disorders as well as in healthy subjects. Among the multiple effects elicited by tDCS on cognitive functions, experimental evidence and clinical findings have highlighted the beneficial impact on long-term memory. Memory deficits occur during physiological aging as well as in neurological and neurodegenerative disorders, including Alzheimer’s disease (AD). In this scenario, non-invasive techniques for memory enhancement, such as tDCS, are receiving increasing attention. The knowledge of molecular mechanisms subtending tDCS effects is of pivotal importance for a more rationale use of this technique in clinical settings. Although we are still far from having a clear picture, recent literature on human and animal studies has pointed to the involvement of synaptic plasticity mechanisms in mediating tDCS effects on long-term memory. Here we review these studies focusing on the neurotrophin “brain-derived neurotrophic factor” (BDNF) as critical tDCS effector.

## Introduction

Transcranial direct current stimulation (tDCS) is a tool effectively modulating motor and cognitive functions in humans. tDCS protocols consist of low amplitude direct currents delivered to human brain via two electrodes placed over the scalp. Depending on electrode positioning (anode or cathode located over the target area), tDCS exerts opposite effects: “anodal” tDCS depolarizes membrane potential and increases excitability in stimulated neurons, whereas “cathodal” tDCS produces neuronal hyperpolarization. Different electrode configurations producing different results are used: (i) unilateral configuration (i.e., one electrode positioned over the target cortical area and the other one over the contralateral supraorbital region or, in some cases, extracephalically); (ii) bilateral configuration (i.e., one electrode positioned over the target cortical area and the other one over the contralateral side); (iii) high-definition tDCS (HD-tDCS), recently developed to improve tDCS spatial focality and based on multiple electrodes (Bikson et al., 2010). In animal models, the unilateral configuration is usually preferred to prevent current bypassing because of the limited space available for two juxtaposed electrodes. This configuration is achieved by positioning the active electrode over the target cortical area and the other one extracephalically, typically over the ventral thorax (Jackson et al., 2016).

Early tDCS studies, mainly focused on motor system, demonstrated that anodal tDCS of the human motor cortex enhanced motor cortex excitability, while cathodal stimulation decreased it (Nitsche and Paulus, 2000; Liebetanz et al., 2002; Lang et al., 2004). tDCS of motor cortex has also been used for the treatment of movement disorders, including dystonia, Parkinson’s disease and stroke (Flöel, 2014). Afterwards, multiple effects of tDCS applied over different brain areas have been described in humans and modulation of brain plasticity has been suggested to account for tDCS after-effects lasting days or weeks (Polanía et al., 2011; Pilato et al., 2012; Kuo et al., 2013; Monte-Silva et al., 2013; Di Pino et al., 2014; **Table 1**). tDCS applied over the dorsolateral prefrontal cortex (DLPFC) induced enhancement of high-order cognitive processes, such as working memory, attention and perception (Coffman et al., 2014), in healthy subjects as well as in patients suffering from neuropsychiatric disorders, including depression and schizophrenia (Bennabi et al., 2014). Furthermore, growing evidence suggests that tDCS has a beneficial impact on long-term memory, that is supported by synaptic plasticity and adult neurogenesis, mechanisms that are both modulated by electromagnetic stimuli (Di Lazzaro et al., 2013; Coffman et al., 2014; Leone et al., 2014, 2015; Podda et al., 2014). Specifically, clinical studies indicated that tDCS (1–2 mA; 5–30 min) of DLPFC or temporal lobe enhances both episodic and semantic memories in humans and that these effects outlast the stimulation period (Marshall et al., 2004, 2005, 2006; Boggio et al., 2009; Hammer et al., 2011; Clark et al., 2012; Falcone et al., 2012; Flöel et al., 2012; Brunyé et al., 2014; Cotelli et al., 2014; Fertonani et al., 2014; Meinzer et al., 2014; Gray et al., 2015; Chua et al., 2017; Schaal et al., 2017). In older subjects, anodal tDCS improves memory to a level comparable with younger controls (Meinzer et al., 2013) and in patients suffering from Alzheimer’s disease (AD) it enhances visual recognition memory for objects (Boggio et al., 2009).

**Table 1:** Studies on neuroplasticity effects of DCS/tDCS in humans and animal models.

Reference	Stimulation protocol	Target area	Model	Effects	Observation time window post-DCS/tDCS
Fritsch et al., 2010	Anodal DCS (10 μA; 15 min) combined with LFS (0.1 Hz)	M1	*DCS of mouse brain slices*	Induction of LTP	30 min
Ranieri et al., 2012	Anodal DCS (200–250 μA; 20 min)	Hippocampus	*DCS of rat brain slices*	Increase of LTP	1–5 h
	Cathodal DCS (200–250 μA; 20 min)	Hippocampus	*DCS of rat brain slices*	Reduction of LTP	1–5 h
Kronberg et al., 2017	Anodal DCS (100–200 μA; 45 s) combined with HFS (20 Hz)	Hippocampus	*DCS of rat brain slices*	LTP: no effects in apical dendrites; increase in basal dendrites	1 h
	Cathodal DCS (100–200 μA; 45 s) combined with HFS (20 Hz)	Hippocampus	*DCS of rat brain slices*	LTP: increase in apical dendrites; no effects in basal dendrites	1 h
	Anodal or cathodal DCS (100–200 μA; 30 min) combined with LFS (0.5 Hz)	Hippocampus	*DCS of rat brain slices*	Attenuation of LTD	1 h
Rohan et al., 2015	Anodal tDCS (100–250 μA; 30 min)	Hippocampus	*tDCS in rats*	Increase of LTP	24 h
Podda et al., 2016	Anodal tDCS (350 μA; 20 min)	Hippocampus	*tDCS in mice*	Increase of LTP	1 week
	Cathodal tDCS (350 μA; 20 min)	Hippocampus	*tDCS in mice*	Reduction of LTP	2–6 h
Nitsche et al., 2003	Anodal tDCS (1 mA; 11–13 min)	M1	*tDCS in humans*	Increase of excitability; No effects in the presence of NMDAR, Na^+^ and Ca^2+^ channel antagonists	1 h
	Cathodal tDCS (1 mA; 9 min)	M1	*tDCS in humans*	Reduction of excitability; No effects in the presence of NMDAR antagonist	1 h
Kuo et al., 2013	Anodal tDCS or HD-tDCS (2 mA; 10 min)	M1	*tDCS in humans*	Increase of excitability	2 h (tDCS); >2 h (HD-tDCS)
	Cathodal tDCS or HD-tDCS (2 mA; 10 min)	M1	*tDCS in humans*	Reduction of excitability	2 h (tDCS); >2 h (HD-tDCS)
Monte-Silva et al., 2013	Anodal tDCS (single session: 1 mA; 13 min)	M1	*tDCS in humans*	Increase of excitability	1 h
	Anodal tDCS (2 sessions: 1 mA; 13 min; time interval of 3–20 min)	M1	*tDCS in humans*	Long-lasting increase of excitability; No long-lasting effects in the presence of NMDAR antagonist	>24 h
	Anodal tDCS (2 sessions: 1 mA; 13 min; time interval of 3–24 h)	M1	*tDCS in humans*	No effects on excitability	
Polanía et al., 2011	Bilateral tDCS (1 mA; 10 min)	Anode over left M1 and cathode over right DLPFC	*tDCS in humans*	Increase of connectivity degree	5 min


Similar results were observed in rodents, in which tDCS (100–350 μA; 20 min) applied over PFC, temporal cortex or hippocampus boosts memories (object-recognition, spatial and fear memories) in both physiological (Binder et al., 2014; Podda et al., 2016; Manteghi et al., 2017; Nasehi et al., 2017) and pathological models, including AD (Yu et al., 2015) and traumatic brain injury (TBI) (Yoon et al., 2016).

Given the constant growth of the elderly population worldwide, memory deficits associated to physiological aging and neurodegenerative disorders are rapidly increasing and, unfortunately, pharmacological strategies are poorly effective to either counteract or delay the onset of memory decline. Therefore, non-pharmacological techniques for memory enhancement, including tDCS, are receiving increasing attention. At the same time, there is urgent need to advance our knowledge of molecular mechanisms subtending neurobiological effects of tDCS for a more rational use of this technique, to maximize its benefits and minimize its potential adverse effects. Studies on animal models have been very useful to provide insights into the cellular and molecular mechanisms underlying tDCS effects on memory. Of note, convergent evidence from human and animal studies points to the neurotrophin “brain-derived neurotrophic factor” (BDNF) as critical determinant of tDCS effects.

Within this frame, here we will review recent literature concerning tDCS effects on explicit memory and the underlying molecular mechanisms, focusing on those involving synaptic plasticity and BDNF. Human and animal studies will be discussed in parallel to highlight the potential of translating information obtained from animal models to clinical practice.

## tDCS Modulation of Synaptic Plasticity-Related Events

The molecular determinants of tDCS-induced enhancement of memory have been recently investigated. However, so far, only few studies, mainly focused on motor cortex, examined cellular and molecular mechanisms enrolled by tDCS in humans. Specifically, a pharmacological study suggested that tDCS-induced changes in motor cortical excitability were mediated by *N*-methyl-D-aspartate receptors (NMDARs) and membrane depolarization (Liebetanz et al., 2002). Nitsche et al. (2003) demonstrated that sodium and calcium channels were also involved in long-term excitability changes induced by tDCS in the human motor cortex.

Magnetic resonance studies on humans also showed a polarity-dependent modulation of neurotransmitter concentration following tDCS. In particular, in both young and old adults, anodal tDCS of the motor cortex induced a significant decrease in γ-aminobutyric acid (GABA) concentration whereas cathodal stimulation led to a significant decrease in glutamate (Stagg et al., 2009; Antonenko et al., 2017). Clark et al. (2011) demonstrated that anodal tDCS applied over the right parietal cortex led to increased Glx (a combination of glutamate and glutamine) and N-acetylaspartate (NAA) in the stimulated area with respect to unstimulated contralateral hemisphere. *In vivo* microdialysis performed on rat models showed that dopamine levels were increased in the striatum following cathodal tDCS and these effects were seen for more than 6 h (Tanaka et al., 2013).

On the basis of these findings and considering that tDCS effects on neuronal excitability and behavior outlasted the stimulation period (Bindman et al., 1964; Reis et al., 2009; Flöel et al., 2012), it was hypothesized that long-lasting changes in synaptic strength, i.e., synaptic plasticity, might be involved in mediating tDCS after-effects (Pilato et al., 2012; Cirillo et al., 2017). Experimental evidence supported this hypothesis by showing that anodal DCS applied to mouse motor cortex slices concomitantly with synaptic activation induced NMDAR-dependent LTP, a well-established form of activity-dependent long-term changes in synaptic efficacy (Malenka and Bear, 2004; Fritsch et al., 2010). Additionally, a consistent body of evidence obtained from *in vitro* and *in vivo* studies indicates that tDCS exerts modulatory effects on LTP and LTD (Ranieri et al., 2012; Rohan et al., 2015; Podda et al., 2016; Kronberg et al., 2017; **Table 1**). In particular, it has been demonstrated that DCS applied to rat hippocampal slices prior to synaptic plasticity induction protocol, modulated LTP in a polarity-dependent manner, with anodal DCS enhancing LTP and cathodal DCS decreasing it. Anodal and cathodal DCS also induced the expression of immediate early genes (IEGs), such as *c-fos* and *zif268*, in the rat hippocampus (Ranieri et al., 2012). Further *in vivo* studies showed that tDCS exerted polarity-dependent modulatory action on hippocampal LTP. In particular, anodal tDCS (100–250 μA; 30 min) applied *in vivo* over the hippocampus of freely moving rats enhanced hippocampal LTP and paired-pulse facilitation. Anodal tDCS effects on hippocampal LTP were intensity-dependent, persisted 24 h after the end of tDCS protocol and were mediated by NMDARs (Rohan et al., 2015).

Parallel studies by Podda et al. (2016) demonstrated that anodal tDCS (350 μA; 20 min) applied over the hippocampus of freely moving mice enhanced hippocampal LTP and this effect was seen up to 1 week after stimulation. Reduced hippocampal LTP was instead observed in slices obtained from mice subjected to cathodal tDCS (Podda et al., 2016). Moreover, it has been shown that DCS applied to rat hippocampal slices concomitantly with synaptic plasticity induction protocol exerts different effects on LTP, likely related to dendritic location. Specifically, in apical dendrites cathodal DCS enhanced hippocampal LTP, while anodal DCS had no significant effects. Conversely, in basal dendrites anodal DCS enhanced LTP while cathodal DCS did not exert significant effects. Both anodal and cathodal DCS reduced hippocampal LTD in apical dendrites, indicating that DCS effects on LTD are not polarity-dependent. The different effects of DCS on LTP have been attributed to modifications in membrane potential occurring in apical and basal dendrites. In particular, cathodal DCS (i.e., cathode closer to CA1 apical dendrites) would hyperpolarize somas and basal dendrites and depolarize apical dendrites, whereas anodal DCS (i.e., anode closer to CA1 apical dendrites) would exert opposite effects (Kronberg et al., 2017). Although the mechanisms underlying the effects of DCS on LTP were not investigated by this study, it can be hypothesized that downstream effectors, including BDNF, are similarly affected in apical and basal dendrites (i.e., where LTP enhancement was observed) receiving depolarizing current.

**FIGURE 1:**
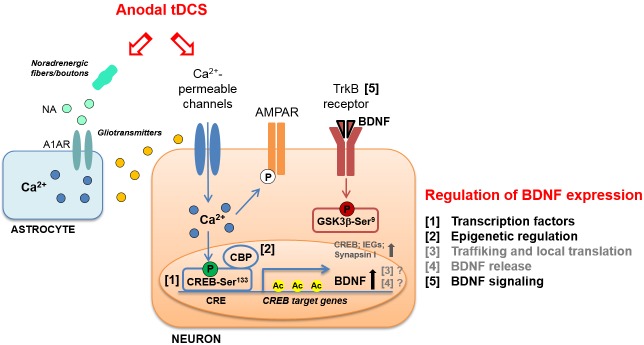
Schematic representation of molecular cascades involved in anodal transcranial direct current stimulation (tDCS)-induced enhancement of synaptic plasticity and memory, in animal models. Experimental evidence suggests that tDCS-induced enhancement of explicit memory is mediated by a molecular cascade including: (i) transient increase in intraneuronal Ca^2+^; (ii) increased activation of CREB by Ser^133^ phosphorylation; (iii) CREB binding to *brain-derived neurotrophic factor (BDNF)* promoter I; (iv) recruitment of CBP; (v) H3K9 acetylation of *BDNF* promoter I by CBP; (vi) persistent increase in BDNF expression; (vii) activation of TrkB receptor; (viii) inhibition of GSK-3β by Ser^9^ phosphorylation. The multiple levels of regulation of BDNF expression are numbered (1–5) and listed on the right; those engaged by anodal tDCS are indicated in black. The same regulatory systems might also be engaged by tDCS to enhance the expression of other plasticity-related genes, such as CREB, synapsin I and IEGs. Increased phosphorylation of AMPAR GluA1 subunit at Ser^831^ in the hippocampus has also been documented following anodal tDCS. Moreover, tDCS was shown to target astrocytes by inducing: (i) noradrenaline (NA) release from noradrenergic fibers or boutons and (ii) NA-induced astrocytic Ca^2+^ elevation thorough the α_1_ adrenergic receptor (A1AR). The Ca^2+^-mediated release of gliotransmitters (glutamate, ATP, D-serine, etc.) from astrocytes might contribute to the enhancement of NMDAR-dependent plasticity induced by tDCS.

Further evidence supports the view that tDCS elicits LTP-like mechanisms, showing that a single session of stimulation (250 μA; 20 min) affects hippocampal α-Amino-3-hydroxy-5-methyl-4-isoxazole propionic acid receptors (AMPARs). In particular, tDCS promotes both translocation of GluA1-containing AMPARs from cytosol to synaptic membrane and GluA1 subunit phosphorylation at Ser^831^ (Stafford et al., 2017; **Figure 1**).

Consistent with the notion that LTP-like plasticity mechanisms are involved in the consolidation of memories (Lynch, 2004), it was found that anodal tDCS of the hippocampus improved spatial and recognition memory in mice, as revealed by Morris Water Maze and Novel Object Recognition tasks. Interestingly, both LTP and memory enhancement induced by anodal tDCS persisted 1 week after stimulation (Podda et al., 2016).

Studies discussed so far on both humans and animal models strongly suggest that memory enhancement induced by different tDCS protocols might be mediated by common molecular mechanisms involving NMDAR-dependent synaptic plasticity events. Afterwards, numerous studies have been performed to further investigate the molecular cascade enrolled by tDCS that might explain long-lasting effects on explicit memory.

## Role of BDNF in tDCS Effects on Memory

Literature reports provided convincing evidence on the role of BDNF in synaptic plasticity, learning and memory (Kowiański et al., 2018). In particular, spatial memory training enhanced the expression of both pro-BDNF and BDNF “tyrosine receptor kinase B” (TrkB) receptors in the hippocampus (Silhol et al., 2007). The activity-dependent increase of BDNF is mediated by NMDAR stimulation and subsequent Ca^2+^ influx. This event leads to activation of the transcription factor “cAMP response-element-binding protein” (CREB), which binds to BDNF promoter, thus triggering BDNF transcription (Zafra et al., 1991; Ghosh et al., 1994; Tao et al., 1998, 2002). It is also well known that BDNF plays a key role in both the early phase and the late phase of LTP (Woo and Lu, 2009).

Given the critical role of BDNF in synaptic plasticity, its involvement in mediating tDCS effects on motor and cognitive functions has been thoroughly investigated. BDNF gene is characterized by several single nucleotide polymorphisms (Liu et al., 2005) and one of these, causing a substitution of valine to methionine at position 66 (Val66Met), has been linked to a reduced responsiveness to tDCS. In particular, Fritsch and collaborators reported that tDCS-induced motor skill enhancement was greater in individuals homozygous for the Val allele than in Met allele carriers (Fritsch et al., 2010). Moreover, in subjects with Val66Met polymorphism cathodal tDCS did not exert any pre-conditioning effect on the response to a subsequent TMS protocol (Cheeran et al., 2008). These effects may be associated to mechanistic differences in the regulation of BDNF expression between Val/Val and Met allele carriers, affecting crucial sites for tDCS action. Indeed, in the hippocampus of BDNF^Met/Met^ mice compared to BDNF^V al/V al^ mice was found: (i) reduced expression of BDNF exon IV and VI transcripts; (ii) increased trimethylation of histone 3 at lysine 27 at *BDNF* promoters V, VI and VIII; (iii) impaired trafficking of BDNF VI transcript to dendrites; (iv) reduced levels of BDNF protein (Mallei et al., 2015). Furthermore, decreased hippocampal volume and impaired hippocampal-dependent explicit memory have been described in BDNF^Met/Met^ subjects (Chaieb et al., 2014).

These studies, which mainly refer to tDCS effects on motor cortex, provide a rationale for further investigating the involvement of BDNF in tDCS-induced modulation of motor and cognitive functions. This issue has been addressed by using animal models. Specifically, it has been demonstrated that potentiation of post-synaptic responses observed in motor cortex slices following DCS was not elicited in slices obtained from BDNF and TrkB mutant mice (Fritsch et al., 2010). Podda et al. (2016) also demonstrated a causal-link between BDNF and tDCS effects on memory by showing that pharmacological inhibition of TrkB receptors with ANA-12 hindered tDCS effects on memory. Likewise, Kim et al. (2017) showed that repetitive anodal tDCS (250 μA; 20 min; once per day for seven consecutive days) applied over the right sensorimotor cortex of healthy rats enhanced mRNA levels of plasticity-associated genes, including *BDNF*, *CREB*, *CaMKII*, *synapsin I*, and IEGs, such as *c-fos* (**Figure 1**).

The role of BDNF in mediating tDCS effects on memory has also emerged from studies on animal models of disease. Indeed, anodal tDCS of the frontal cortex rescued short-term memory deficits in spontaneous hypertensive rats (SHR) that are the most widely used animal models of attention-deficit/hyperactivity disorder. tDCS also increased dopamine levels in the striatum and hippocampus of SHR rats and, importantly, enhanced BDNF levels in the striatum (Leffa et al., 2016). Moreover, it has been reported that anodal tDCS applied over the motor perilesional cortex of rat models of TBI for 2 weeks post-injury improved spatial memory and increased BDNF levels (Yoon et al., 2016).

As a growing body of evidence implicates BDNF in tDCS effects on synaptic plasticity and memory under both physiological and pathological conditions, the BDNF signaling pathway activated by tDCS has been put into focus.

## Insights Into the Regulation of BDNF Expression by tDCS

BDNF expression is highly regulated at several levels. The structure of *BDNF*, consisting of eight 5′ non-coding exons and a 3′ coding exon (i.e., exon IX) under the control of different promoters, allows a temporal and spatial regulation of BDNF expression by multiple stimuli. Among the different stimuli regulating BDNF expression, neuronal activity has been described as the most potent one (Aid et al., 2007; Chen and Chen, 2017). In addition to the binding of sequence-specific transcription factors to different promoters, transcription of *BDNF* is modulated by stimulus-dependent changes in chromatin structure, i.e., epigenetic modifications and, in particular, histone acetylation at *BDNF* promoter I has been shown to affect LTP and long-term memories (Alarcón et al., 2004). Other *BDNF* regulation systems are proteolytic processing, binding to distinct receptors and activation of different signaling cascades (Benarroch, 2015; **Figure 1**).

As for the intracellular messengers linking tDCS to the downstream molecular cascades leading to BDNF regulation, involvement of increased Ca^2+^ levels has been documented. Indeed, tDCS effects have been associated with membrane depolarization-dependent increases in intracellular Ca^2+^ levels via NMDAR and voltage-gated calcium channel activation (Pelletier and Cicchetti, 2014). Of note, increased Ca^2+^ levels could initiate molecular pathways leading to enhanced acetylation via CREB/CBP (Kornhauser et al., 2002). Activation of Ca^2+^ signaling by tDCS has also been reported in cortical astrocytes as a result of increased noradrenaline release that might conceivably contribute to tDCS-induced neural plasticity and epigenetic modifications (Monai and Hirase, 2016; Monai et al., 2016; **Figure 1**).

Interestingly, our recent study showed that tDCS induced: (i) enhanced phosphorylation of CREB at Ser^133^; (ii) increased binding of activated CREB to the *BDNF* promoter I; (iii) the recruitment to *BDNF* promoter I of the transcriptional coactivator CBP, which acts as a histone acetyltransferase (Podda et al., 2016). tDCS-induced epigenetic modifications at *BDNF* promoter I consisted in increased acetylation of histone 3 at lysine 9 (H3K9ac) (Podda et al., 2016; **Figure 1**). This event has been considered the main molecular mechanism whereby anodal tDCS enhanced BDNF exon I and IX mRNA and protein expression up to 1 week after stimulation. As a proof-of principle, we treated mice with the p300/CBP HAT inhibitor, curcumin (Zhu et al., 2014), before subjecting them to tDCS and demonstrated that this treatment occluded tDCS effects on learning and memory, LTP and BDNF expression.

The BDNF downstream signaling pathways activated by tDCS have been also investigated. In particular, glycogen synthase kinase-3β (GSK-3β) is a key substrate of TrkB receptors and is mostly involved in synaptic plasticity (Peineau et al., 2008). Data provided by Podda et al. (2016) demonstrated that tDCS enhanced GSK-3β phosphorylation at Ser^9^ (pGSK-3βSer^9^), which is known to promote GSK-3β inhibition resulting in LTP enhancement (**Figure 1**).

## Conclusion

In the last few years, the impact of tDCS on cognitive domains, especially on memory, has been thoroughly studied. In this context, animal models have provided several insights into neuroplasticity changes and molecular events occurring following tDCS that fit well with the results of clinical studies. In particular, it has been demonstrated that BDNF has a crucial role in mediating the beneficial effects of tDCS on explicit memory. Recent findings also showed increased acetylation levels at *BDNF* promoter I soon after as well as 1 week after tDCS, thus providing a possible molecular mechanism responsible for long-lasting tDCS effects.

Data accrued so far about tDCS effects on memory clearly indicate the need of more in-depth analyses of tDCS action on cognitive functions by combining clinical studies with basic research that, despite the limits of animal models, has the advantage of providing mechanistic insights on effects observed in humans. The results of these studies might pave the way to the identification of promising strategies for personalized medicine to treat cognitive impairment in neuropsychiatric disorders.

Toward this effort, it should be pointed out that, whereas most studies on explicit memory in humans have been performed targeting DLPFC, the majority of data from animal models refers to the hippocampus. Therefore, one possible direction of future studies is to determine the impact of tDCS in tuning hippocampal-prefrontal interactions given the well-established role of this circuit in memory and high-order cognitive functions (Sigurdsson and Duvarci, 2016).

## Author Contributions

SC, MVP, and CG conceived the review and reviewed the literature. SC wrote the manuscript. MVP and CG revised the manuscript. All authors finalized the manuscript and approved the current version of this paper.

## Conflict of Interest Statement

The authors declare that the research was conducted in the absence of any commercial or financial relationships that could be construed as a potential conflict of interest.
